# Cardioprotective Effects of a Novel Hydrogen Sulfide Agent–Controlled Release Formulation of S-Propargyl-Cysteine on Heart Failure Rats and Molecular Mechanisms

**DOI:** 10.1371/journal.pone.0069205

**Published:** 2013-07-09

**Authors:** Chengrong Huang, Juntao Kan, Xu Liu, Fenfen Ma, Ba Hieu Tran, Yunzeng Zou, Shujun Wang, Yi Zhun Zhu

**Affiliations:** 1 School of Pharmacy, Fudan University, Shanghai, Shanghai, China; 2 School of Pharmacy & Institutes of Biomedical Sciences, Fudan University, Shanghai, Shanghai, China; 3 Zhongshan Hospital & Institutes of Biomedical Sciences, Fudan University, Shanghai, Shanghai, China; 4 School of Pharmacy, Shenyang Pharmaceutical University, Shenyang, Liaoning, China; The Chinese University of Hong Kong, Hong Kong

## Abstract

**Objective:**

Heart failure (HF) is one of the most serious diseases worldwide. S-propargyl-cysteine (SPRC), a novel modulator of endogenous hydrogen sulfide, is proved to be able to protect against acute myocardial ischemia. In order to produce more stable and sustainable hydrogen sulfide, we used controlled release formulation of SPRC (CR-SPRC) to elucidate possible cardioprotective effects on HF rats and investigate involved mechanisms on apoptosis and oxidation.

**Methods:**

Left coronary artery was occluded to induce HF model of rat. The survival rats were randomly divided into 7 groups after 24 hours and treated with drugs for 6 weeks. Echocardiographic indexes were recorded to determine cardiac function. TTC staining was performed to determine infarct size. Plasmatic level of hydrogen sulfide was detected by modified sulfide electrode. Activity of enzyme and expression of protein were determined by colorimetry and Western blot, respectively.

**Results:**

The cardioprotective effects of CR-SPRC on HF rats were confirmed by significant reduction of infarct size and improvement of cardiac function, with better effects compared to normal SPRC. CR-SPRC modulated antioxidant defenses by preserving levels of GSH, CAT and SOD and reducing CK leakage. In addition, CR-SPRC elevated ratio of Bcl-2/Bax and inhibited activity of caspases to protect against myocardial apoptosis. The cardioprotective effects of CR-SPRC were mediated by hydrogen sulfide.

**Conclusions:**

All experiment data indicated cardioprotective effects of CR-SPRC on HF rats. More importantly, CR-SPRC exerted better effects than normal SPRC in all respects, providing a new perspective on hydrogen sulfide-mediated drug therapy.

## Introduction

Heart failure (HF) is a condition in which the heart can no longer pump sufficient blood to meet the need of the body [1]. More than 23 million people around the world are suffering from this disease, causing it to be the most serious health issue in both developed and developing countries. Many factors such as ischemic heart disease, hypertension, diabetes, dyslipidemia and smoking can increase the risk for development of HF [2]. Although some drugs, including diuretics, vasodilators, and digoxin, have been used in treatment of HF for many years, their side effects cannot be ignored [3].

As the third gasotransmitter, hydrogen sulfide has been investigated more and more popularly these years [4]. Although discovered as endogenous neuromodulator in the last century [5], hydrogen sulfide is indeed famous for its protective effects in the cardiovascular system [6]. Besides as an acknowledged K_ATP_ opener [7], hydrogen sulfide has been proved to play many roles in protecting against cardiovascular diseases such as hypertension [8], atherosclerosis [9], acute myocardial ischemia [10], [11] and chronic heart failure [12].

S-propargyl-cysteine (SPRC, reported also as ZYZ-802), a novel modulator of endogenous hydrogen sulfide, is developed to address the fatal problem of the administration of hydrogen sulfide. Unlike the gas itself and exogenous donor of hydrogen sulfide, SPRC is designed to effect through promoting the activity of cystathionine-γ-lyase (CSE), a metabolic enzyme that could generate endogenous hydrogen sulfide [4]. In order to produce more stable and sustainable hydrogen sulfide, we developed controlled release formulation of SPRC (CR-SPRC) by solid dispersion technique with Eudragit (R) RS30D as carrier.

In the previous studies, we proved SPRC and two other cysteine analogues could protect against acute myocardial ischemia by reducing the deleterious effects of oxidative stress through modulating the endogenous hydrogen sulfide [13]. In this work, we elucidated the cardioprotective effects of CR-SPRC on HF rats and investigated the involved mechanisms on apoptosis and oxidation, offering some insights into the long-term and sustained releasing drug therapy of hydrogen sulfide.

## Materials and Methods

### Experimental Animals

Adult male Sprague-Dawley (SD) rats weighing 200–220 g were purchased from Sippr-bk Experimental Animal Center (Shanghai, China), and kept under standard conditions of animal room (temperature 25°C; humidity 55–60%). All animal care and experimental protocols complied with the Animal Management Rules of the Ministry of Health of the People’s Republic of China and approved by the local ethical committee of Fudan University. All surgery was performed under anesthesia of chloral hydrate or pentobarbital sodium, and all efforts were made to ameliorate animal suffering.

### Drugs and Pharmaceutical Preparation

Propagylglycine (PAG, a CSE inhibitor) was purchased from Intechem Technology (Shanghai, China), and digoxin was purchased from Sine Pharmaceutical Co. (Shanghai, China). SPRC was synthesized from the reaction of L-cysteine with propargyl bromide and then purified by recrystallization from ethanol-water mixture (99%). CR-SPRC was prepared by solid dispersion technique as described previously [14]. In brief, 0.1 g of SPRC was dissolved in Eudragit (R) RS30D (Rohm Pharma, Weiterstadt, Germany) and placed in a round-bottomed flask which kept constant magnetic stirring at midrange rotation speed and maintained at 80°C in a thermostat-controlled water bath. The solvent was then removed and cooled down to −20°C immediately. After heat treating at 50°C for 4 h, the co-precipitates were desiccated, pulverized, and sieved through 100 µm mesh. CR-SPRC was dissolved in 0.5% sodium carboxymethylcellulose (CMC-Na) for drug treatment.

### Induction of Chronic Heart Failure Model

After anesthetized by 7% chloral hydrate (350 mg•kg^−1^, intraperitoneal injection), SD rats were connected to an electrocardiograph (ECG) recorder and treated by tracheal intubation, then subjected to left coronary artery ligation (n = 110) or sham surgery (n = 10). The left coronary artery was ligated permanently to induce HF model of rat as described previously [11], [12]. In brief, after cutting off the third rib, the left descending anterior coronary was ligated 2–3 mm near its origin with a 6.0 silk thread. Successful ligation was verified by the color change immediately in the ischemic area (anterior ventricular wall and the apex) and the occurrence of arrhythmias (ST-segment elevation). The chest was closed in layers and the skin was sterilized with povidone iodine. 24 hours after the surgery, the survival rate was 69%. Sham-operated animals were performed by thoracotomy to open the pericardium only, with no ligation around the left anterior descending artery.

### Experimental Design and Drug Administration

After 24 hours, the survival rats were randomly divided into seven groups: (1) sham group (n = 10); (2) HF group (n = 14); (3) SPRC-treated HF group (30 mg·kg^−1 ^day^−1^) (n = 12); (4) CR-SPRC-treated HF group (30 mg·kg^−1 ^day^−1^) (n = 13); (5) CR-SPRC plus PAG-treated HF group (30 mg·kg^−1 ^day^−1^+10 mg·kg^−1 ^day^−1^) (n = 14); (6) PAG-treated HF group (10 mg·kg^−1 ^day^−1^) (n = 16) (7) Digoxin-treated HF group (0.2 mg·kg^−1 ^day^−1^) (n = 11). Besides PAG by intraperitioneal injection, all the other drugs were given by intragastric administration. During the treatment period of 6 weeks, body weight of the rats was measured every two days, and the dosage of drugs was adjusted according to the body weight. The death of animals was recorded every day.

### Echocardiography

Six weeks after the surgery, rats were anesthetized with pentobarbital sodium (30 mg·kg^−1^) and placed on a heating pad. Cardiac function of the rats was dynamically evaluated by echocardiography using Vevo770 (Visual Sonics Inc., Toronto, Canada) with a 716 probe. The transducers with frequency of 17.5-MHz for ventricular structure provided spatial resolutions. Left ventricular internal dimension in systole (LVIDs), left ventricular internal dimension in diastole (LVIDd), left ventricular anterior wall in systole (LVAWs), left ventricular anterior wall in diastole (LVAWd), left ventricular posterior wall in systole (LVPWs) and left ventricular posterior wall in diastole (LVPWd) were obtained from the M-mode tracings, while other parameters such as left ventricular volume in systole (LVs), left ventricular volume in diastole (LVd), ejection fraction (EF) and fractional shortening (FS) were derived automatically by the High-Resolution Electrocardiograph system.

### Determination of Infarct Size

Infarct size was determined by 1% triphenyltetrazolium chloride (TTC) staining. After echocardiography measurements, animals were sacrificed and the hearts were excised immediately, and then stored at −80°C for freeze after PBS washed. Each heart was cut manually into six to eight transverse slices. After dipping in TTC solution at 37°C for 30 minutes, these slices were flushed with saline and then fixed in 4% paraformaldehyde for 30 minutes. Next, the slices were placed on a glass slide and photographed by digital camera, using the ImageJ software (NIH, Boston, MA) to analyze.

### Histopathology Analysis of Myocardial Fibrosis

Myocardial fibrosis was determined by commercially available Masson's trichrome staining kit (Yuanye Biotech, Shanghai, China). Animals were sacrificed and the hearts were excised immediately, and then fixed in 4% paraformaldehyde. Each heart was embedded in paraffin, and cut into sections (5 µm thickness). The sections were stained using Masson’s kit according to the manufacturer’s instruction, and photographed by digital camera, using the ImageJ software (NIH, Boston, MA) to analyze.

### Sensitive Sulphur Electrode Assay

Plasmatic level of hydrogen sulfide was detected after drug treatment for 6 weeks using ISO-H2S-2 sensor (World Precision Instruments, Sarasota, FL). In brief, the collected blood was treated with heparin sodium and centrifuged to get plasma. Subsequently, 20 µl of the plasma was pipetted into 4 ml PBS (PH7.2, 0.05 mol·L^−1^) to be detected by sensitive sulphur electrode. Each sample was detected three times.

### Cell Damage and Oxidative Stress Assay

The measurement of creatine kinase (CK), glutathione(GSH), catalase (CAT) and superoxide dismutase (SOD) in the border zone of infarcted ventricular myocardium was conducted by using commercially available kits according to the manufacturer’s instructions (Jiancheng Bioengineering Institute, Nanjing, China).

### Determination of Caspases

Activity of caspase 3 and caspase 9 in the border zone of infarcted ventricular myocardium was determined by colorimetric assay using a microplate reader at 400 nm. The assay kits were purchased from Biovision (Milpitas, CA).

### Western Blot

The fresh ventricular tissue in infarct area was homogenized by a rotor-stator homogenizer in ice-cold RIPA buffer (Pierce, Pittsburgh, PA). After boiling with loading buffer (Fermentas, Glen Burnie, MD), denatured proteins were separated in SDS-PAGE gel, and transferred onto PVDF membrane. The membrane was blocked with nonfat milk, followed by incubation with primary antibody of Bax, Bcl-2 and CSE (Abcam, Cambridge, MA) at 4°C overnight. HRP-conjugated secondary antibody (Kangchen Bio-tech, Beijing, China) was used to incubate the membrane for another 1 hour the next day. SuperSignal West Pico Chemiluminescent Substrate (Pierce, Pittsburgh, PA) was poured on the membrane to develop the band captured by FluorChem Image System (Alpha Innotech, Santa Clara, California).

### Statistical Analysis

All values were presented as means ± standard deviations. One-way analysis of variance (ANOVA) was used to examine statistical comparisons among groups. Two-tailed Student’s *t* test was used to examine statistical significance of differences between two groups. Kaplan-Meier survival curves were compared by use of a log-rank test. A difference with *P*-value less than 0.05 was considered statistically significant.

## Results

### CR-SPRC Improved Survival of HF Rats

**Figure 1:**
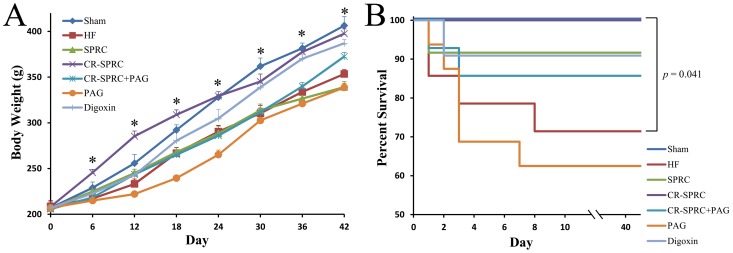
Effects of CR-SPRC on body weight and survival rate of HF rats. (A) Body weight curve. Data were presented as means ± standard deviations (n = 10–16). ^*^ *P*<0.01 HF versus sham, CR-SPRC versus HF, CR-SPRC versus SPRC, CR-SPRC+PAG versus CR-SPRC. (B) Kaplan–Meier survival curve. *P* = 0.041 CR-SPRC versus HF.

Unlike normal SPRC, CR-SPRC did not slow down the growth of body weight of HF rats compared with that in sham group (Figure 1A). Furthermore, intragastric administration of CR-SPRC kept the HF rats survive all 6 weeks (13/13) (Figure 1B). On the contrary, the survival rate of normal HF rats was 71% (10/14), with a significant higher mortality compared with that in CR-SPRC-treated group (*P*<0.05). The data of body weight and survival rate showed that CR-SPRC could be a potential drug without obvious signs of toxicity on trial animals.

### CR-SPRC Improved Cardiac Function of HF Rats

**Figure 2:**
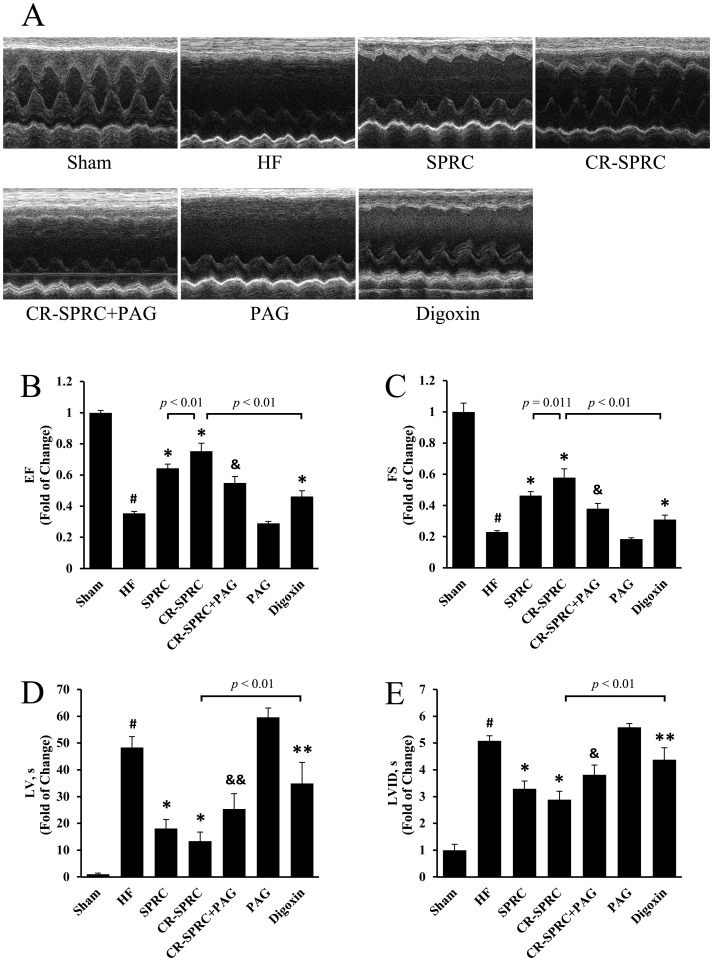
CR-SPRC improved cardiac function of HF rats. (A) Representative echocardiographic records obtained from a short-axis mid-ventrical view of hearts of the HF rats. (B), (C), (D) and (E) were statistical analysis of the data obtained or derived from original echocardiographic records. Data were presented as means ± standard deviations (n = 6). ^#^ *P*<0.01 versus sham, ^*^ *P*<0.01 versus HF, ^**^ *P*<0.05 versus HF, ^&^ *P*<0.01 versus CR-SPRC, ^&&^ *P*<0.05 versus CR-SPRC.

In normal HF rats, permanent ligation of left coronary artery made the cardiac function injured, with much lower EF than that in sham group (34.20±1.17% vs. 96.40±1.51%; *P*<0.01) (Figure 2, Table 1). CR-SPRC preserved the cardiac function (EF) effectively (72.56±4.94% vs. 34.20±1.17%; *P*<0.01), with better effects than normal SPRC and positive drug digoxin (72.56±4.94% vs. 62.01±2.62% and 44.59±3.66%; *P*<0.01). CR-SPRC significantly decreased the elevated LV and LVID caused by HF (87.08±22.06 µl vs. 314.95±26.20 µl and 4.36±0.48 mm vs. 7.68±0.29 mm; *P*<0.01). In contrast to LVAW and LVPW in normal HF group, those in CR-SPRC-treated group showed obvious improvement (2.14±0.32 mm vs. 1.17±0.36 mm and 3.88±0.22 mm vs. 2.72±0.41 mm; *P*<0.01). It was demonstrated that CR-SPRC could reduce the enlarged volume of left ventricle caused by HF as well as increase the thickness of ventricular wall.

**Table 1:** Echocardiographic analysis of cardiac function.

Parameters	Sham (n = 6)	HF (n = 6)	SPRC (n = 6)	CR-SPRC (n = 6)	CR-SPRC+PAG (n = 6)	PAG (n = 6)	Digoxin (n = 6)
EF, %	96.40±1.51	34.20±1.17#	62.01±2.62*	72.56±4.94*	52.93±3.99&	28.06±1.21	44.59±3.66*
FS, %	74.74±4.28	17.18±0.64#	34.60±1.99*	43.24±4.31*	28.37±2.53&	13.85±0.64	23.17±2.16*
LVd, µl	175.22±27.09	478.39±34.92#	311.23±61.38*	315.54±42.71*	349.07±53.67	539.55±25.25	408.35±74.56
LVs, µl	6.51±3.11	314.95±26.20#	117.98±22.10*	87.08±22.06*	165.41±37.23&&	388.29±22.51	227.50±51.46**
LVIDd, mm	5.92±0.42	9.27±0.31#	7.62±0.69*	7.68±0.47*	8.03±0.56	9.79±0.21	8.61±0.72
LVIDs, mm	1.51±0.33	7.68±0.29#	4.98±0.43*	4.36±0.48*	5.76±0.56&	8.44±0.22	6.62±0.67**
LVAWd, mm	1.90±0.22	1.02±0.34#	1.07±0.16	1.35±0.20	1.19±0.11	0.78±0.10	1.06±0.29
LVAWs, mm	3.54±0.16	1.17±0.36#	1.60±0.25	2.14±0.32*	1.56±0.32&&	0.78±0.15	1.49±0.28
LVPWd, mm	2.11±0.17	1.98±0.04	2.22±0.19**	2.13±0.23	2.15±0.23	2.10±0.37	2.13±0.23
LVPWs, mm	3.58±0.29	2.72±0.41##	3.40±0.67	3.88±0.22*	3.29±0.25&&	2.82±0.33	3.04±0.19

Data were presented as means ± standard deviations.

# *P*<0.01 versus sham,

## *P*<0.05 versus sham,

* *P*<0.01 versus HF,

** *P*<0.05 versus HF,

& *P*<0.01 versus CR-SPRC,

&& *P*<0.05 versus CR-SPRC.

### CR-SPRC Reduced Infarct Size and Myocardial Fibrosis of Left Ventricle

**Figure 3:**
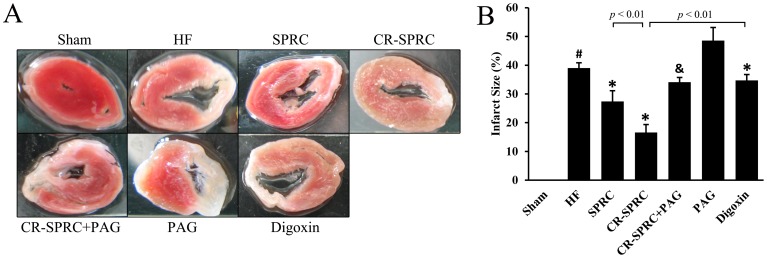
CR-SPRC reduced infarct size of left ventricle. (A) Representative photograph of infarct size which was determined by 1% triphenyltetrazolium chloride (TTC) staining. (B) Statistical analysis of infarct size. Data were presented as means ± standard deviations (n = 5–8). ^#^ *P*<0.01 versus sham, ^*^ *P*<0.01 versus HF, ^&^ *P*<0.01 versus CR-SPRC.

**Figure 4:**
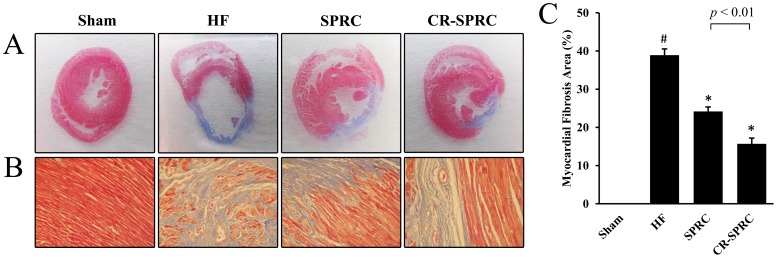
CR-SPRC ameliorated myocardial fibrosis of left ventricle. (A) Representative photograph of myocardial fibrosis which was determined by Masson's trichrome staining. (B) Representative high-magnification microphotograph of Masson-stained sections from border zone in indicated groups in (A). Magnification: ×400. (C) Statistical analysis of myocardial fibrosis. Data were presented as means ± standard deviations (n = 4–6). ^#^ *P*<0.01 versus sham, ^*^ *P*<0.01 versus HF.

Permanent ligation of left coronary artery caused the ischemia of left ventricular myocardium, followed by serious infarct (Figure 3). CR-SPRC significantly reduced the infarct size in the left ventricle compared with that in normal HF group (16.61±2.73% vs. 39.01±1.85%; *P*<0.01), and showed better effects than normal SPRC and positive drug digoxin (16.61±2.73% vs. 27.38±3.74% and 34.70±2.08%; *P*<0.01). Besides, myocardial fibrosis was also found to be substantially ameliorated in CR-SPRC-treated HF rats compared with that in normal HF group (15.69±1.49% vs. 38.92±1.63%; *P*<0.01) (Figure 4).

### The Cardioprotective Effects of CR-SPRC were Mediated by Hydrogen Sulfide

**Figure 5:**
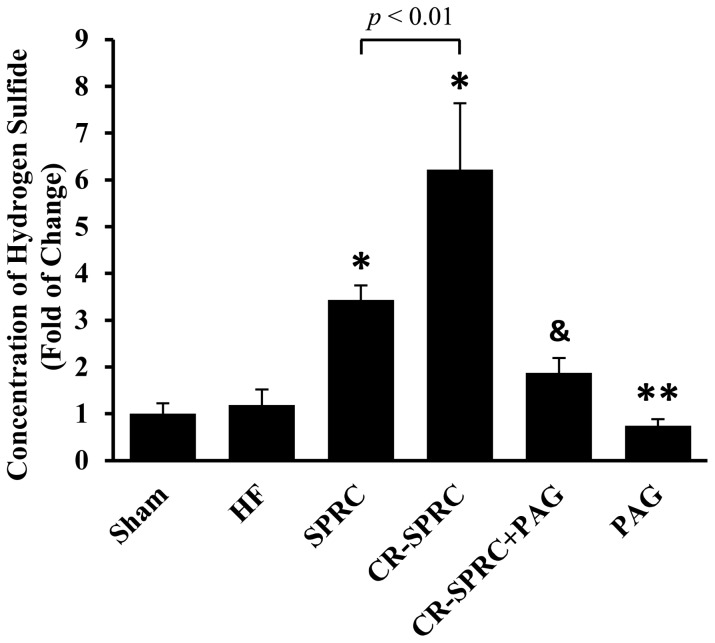
CR-SPRC elevated the level of hydrogen sulfide. The concentration of hydrogen sulfide in plasma was detected by modified sulfide electrode. Data were presented as means ± standard deviations (n = 6). ^*^ *P*<0.01 versus HF, ^**^ *P*<0.05 versus HF, ^&^ *P*<0.01 versus CR-SPRC. All experiments repeated at least 3 times.

**Figure 6:**
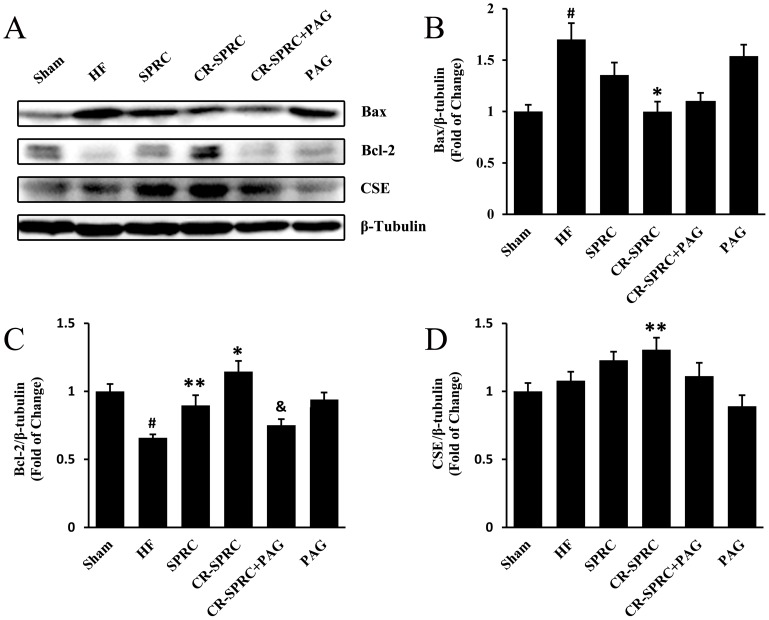
Effects of CR-SPRC on expression of proteins. (A) The expression of Bax, Bcl-2 and CSE were detected by Western blot. β-Tubulin was used as a loading control. (B), (C) and (D) were statistical analysis of (A). Data were presented as means ± standard deviations (n = 5–6). ^#^ *P*<0.01 versus sham, ^*^ *P*<0.01 versus HF, ^**^ *P*<0.05 versus HF, ^&^ *P*<0.01 versus CR-SPRC. All experiments repeated at least 3 times.

We determined the concentration of hydrogen sulfide in plasma to find that CR-SPRC significantly elevated the level compared with that in normal HF group (6-fold), with stronger promoting effect than normal SPRC (*P*<0.01) (Figure 5). In addition, CR-SPRC also promoted the expression of CSE by 21.16% in left ventricle (Figure 6A, D). In order to investigate the possible role of hydrogen sulfide-mediated mechanism in cardioprotection of CR-SPRC, we added PAG, a CSE inhibitor, to treat the animals. It was shown that administration of CR-SPRC plus PAG abolished the stimulative effect of CR-SPRC on hydrogen sulfide level in plasma, and meanwhile, single administration of PAG also lowered the level of hydrogen sulfide in HF rats (Figure 5). Furthermore, treatment of CR-SPRC plus PAG also abolished the positive effects of CR-SPRC on preservation of survival rate (Figure 1), improvement of cardiac function (Figure 2) and reduction of infarct size (Figure 3).

### CR-SPRC Preserved Oxidative Stress and Prevented Cell Damage

**Figure 7:**
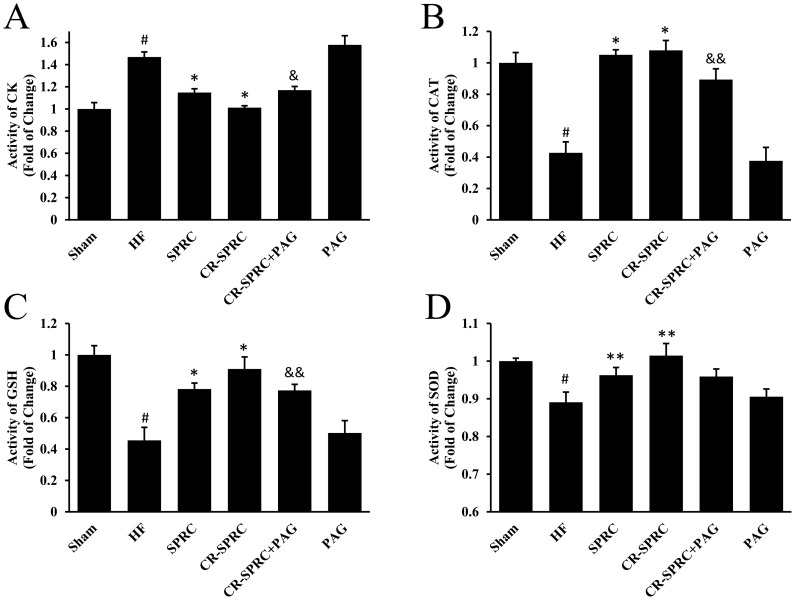
CR-SPRC preserved oxidative stress and prevented cell damage. The levels of CK (A), CAT (B), GSH (C) and SOD (D) in tissue extract of ventricular myocardium were determined by colorimetry, and statistically analyzed. Data were presented as means ± standard deviations (n = 5–6). ^#^P<0.01 versus sham, ^*^P<0.01 versus HF, ^**^P<0.05 versus HF, ^&^P<0.01 versus CR-SPRC, ^&&^P<0.05 versus CR-SPRC. All experiments repeated at least 3 times.

Antioxidant defensive molecules such as CAT, GSH and SOD were decreased in ventricular myocardium of normal HF rats by 57.29%, 54.46%, 10.92%, respectively, which might cause leakage of CK from the damaged cardiomyocytes (Figure 7). CR-SPRC effectively preserved CAT, GSH and SOD at a normal level and prevented the CK leakage. However, administration of CR-SPRC plus PAG could abolish the protective effects of CR-SPRC to a certain extent. In addition, similar results could be also observed in plasma (Figure S1).

### CR-SPRC Protected against Myocardial Apoptosis

**Figure 8:**
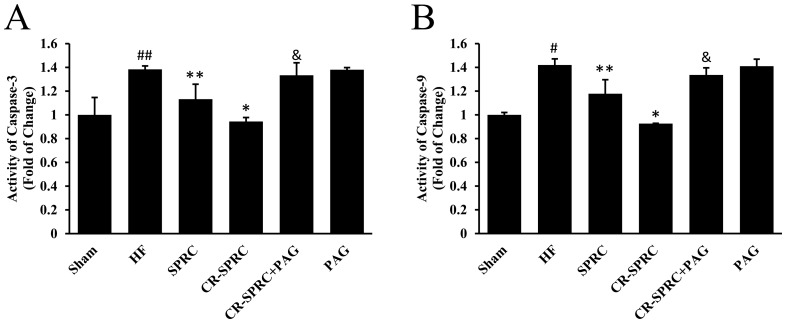
CR-SPRC inhibited activity of caspases. The levels of caspase 3 and caspase 9 in tissue extract of ventricular myocardium were determined by colorimetry, and statistically analyzed. Data were presented as means ± standard deviations (n = 5–6). ^#^ *P*<0.01 versus sham, ^##^ *P*<0.05 versus sham, ^*^ *P*<0.01 versus HF, ^**^ *P*<0.01 versus HF, ^&^ *P*<0.01 versus CR-SPRC. All experiments repeated at least 3 times.

In HF rats, ischemia caused by ligation of left coronary artery induced expression of Bax and reduced expression of Bcl-2 (Figure 6), then triggered the activity of caspase 9 and caspase 3 (Figure 8, S2). CR-SPRC could increase the level of Bcl-2 by 73.91% but decrease the level of Bax, caspase 3 and caspase 9 by 41.31%, 31.74%, 34.76%, respectively, protecting against myocardial apoptosis. Administration of CR-SPRC plus PAG could abolish the influence of CR-SPRC on Bcl-2, caspase 3 and caspase 9, but with limited effects on Bax. In a separated *in vitro* experiment, we also confirmed the anti-apoptotic effects of SPRC in ischemic condition using an H9c2 cell line (Figure S3).

## Discussion

Since the last two decades, the research of hydrogen sulfide on biomedicine has been popular all over the world. The desires of humans to discover hydrogen sulfide originate from the natural property of this amazing gas. It is produced in human body, and regulates the physiological activity to keep homeostasis. The therapeutic potential of hydrogen sulfide is great [15] though the limitation is still obvious. As a small gas molecule, hydrogen sulfide can be easily released and diffused, which makes it difficult for administration. It was reported that exogenous hydrogen sulfide was administrated by infusion in drinking water [16], intracardiac injection [12] or intraperitoneally injection [17] to treat HF. But the biggest problem is the difficulty to grasp the exact dosage. Hydrogen sulfide escapes easily, making the concentration in vehicle falling constantly. However, we are not free to elevate the initial concentration, which could cause intoxication to trial animal or subject. Administration of endogenous hydrogen sulfide through genetic therapy was proved with affirmative effects [12] but still with unknown potential genetic damage. Therefore, targeting critical metabolic enzyme to modulate the endogenous hydrogen sulfide may be a safe and effective direction of hydrogen sulfide-mediated drug therapy in the future.

CSE is one of the metabolic enzymes that could produce endogenous hydrogen sulfide in human and many other mammalian bodies. We did hundreds of docking tests to screen potential small molecules with affinity for CSE through computer aided drug design (CADD). Finally, we focused on a natural compound, S-allyl-cysteine (SAC), which is extracted from aged garlic and showed antioxidant activity. And then we designed a novel cysteine analogue, SPRC, which behaved better than SAC in docking tests. In the later research, we proved SPRC showed significant cardioprotection on acute myocardial ischemia through modulating the production of endogenous hydrogen sulfide [13], [18]. In order to produce more stable and sustainable hydrogen sulfide, we used Eudragit (R) RS30D as carrier to get CR-SPRC by solid dispersion technique. Through *in vitro* release profile and *in vivo* pharmacokinetics study, it was testified that poorly water-soluble polymeric carrier Eudragit could effectively prolong the release of SPRC as well as improve the bioavailability of it (Figure S4, S5). Therefore, CR-SPRC subsequently prolonged the release of endogenous hydrogen sulfide, and kept its level stable for longer period (data not shown). Besides, CR-SPRC reduced the concentration peak of hydrogen sulfide (data not shown), causing organism possibly suffered lower toxicity from hydrogen sulfide (Figure 1). Considering the long-term therapy of hydrogen sulfide to HF, we chose CR-SPRC as a potential drug.

Some research groups have reported the protective effects of hydrogen sulfide on HF animals before [12], [16], [17]. Calvert and fellows researched ischemia-induced HF in mice to find single injection, instead of daily, of hydrogen sulfide reduced infarct size but could not improve structure and function of left ventricle [12]. That demonstrated the treatment of HF was a long-term process. In this work, we treated HF rats with CR-SPRC by intragastric administration for 6 weeks to discover CR-SPRC not only reduced infarct size but also improved cardiac function of HF rats. More importantly, compared with normal SPRC, CR-SPRC showed obviously better effects on HF rats. The main cause might be sustained release of SPRC kept its level stable during a period of time. On the contrary, in an experiment of acute myocardial ischemia, CR-SPRC showed little significant better effects than normal SPRC (data not shown). It might be concluded sustained release of SPRC was more potent in the long-term therapy.

In order to make sure CR-SPRC indeed act through hydrogen sulfide-mediated mechanism, we determined the expression of CSE in myocardial tissue and concentration of hydrogen sulfide in plasma to confirm the critical role of hydrogen sulfide in this process. It was shown that CR-SPRC kept the CSE in myocardial tissue and hydrogen sulfide in plasma to a higher level than did normal SPRC. Besides, the addition of PAG abolished promoted expression of CSE and elevated level of hydrogen sulfide induced by CR-SPRC. Furthermore, preserved body weight and survival rate, reduced infarct size and improved cardiac function were all abolished by addition of PAG. Even administration of PAG alone could decrease the level of hydrogen sulfide in plasma, causing a worse condition than that in normal HF group.

It was found that oxidative stress was elevated in the patients with HF [19], [20], and treatment of vitamin E could ameliorate the development of HF [21]. Therefore release of oxidative stress and reinforcement of antioxidant defense should be considered for treatment of HF [22]. There was evidence that hydrogen sulfide protected neurons from oxidative stress by promoting the production of antioxidant glutathione [23]. In another paper, Calvert and fellows clarified Nrf2, a transcription factor that regulated gene expression of a lot of antioxidants, was involved in hydrogen sulfide-mediated protection on ischemic heart [24]. In this work, we demonstrated as an endogenous modulator of hydrogen sulfide, CR-SPRC indeed preserved the level of antioxidant molecules such as CAT, SOD and GSH, and also prevented the leakage of metabolic enzymes such as CK by keeping the integrity of cardiomyocyte membrane. And the effective effects of CR-SPRC could be abolished by addition of PAG. It could be concluded that the hydrogen sulfide produced by CR-SPRC in myocardium might act as an antioxidant modulator to keep the balance of oxidative stress which associated with the development of HF.

It was also shown that apoptosis occurred in patients with end-stage cardiomyopathy and might contribute to HF [25]. Therefore, antiapoptotic therapy was another direction we focused on to treat HF [26]. Previous studies disclosed hydrogen sulfide protected cardiomyocytes from ischemia-induced apoptosis by preservation of mitochondrial function [10], [17] and preventing GSK-3β-dependent opening of mPTP [27]. In this work, we also confirmed antiapoptotic effects of CR-SPRC on myocardium of HF rats were associated with hydrogen sulfide-mediated modulation through elevating the ratio of Bcl-2/Bax and inhibiting the activity of caspases. However, many papers reported oxidative stress induced apoptosis in cultured cardiac myocytes or myocardial tissue [28], thus we still believed antioxidant effects of CR-SPRC mediated by hydrogen sulfide should play a more prominent role in cardioprotection of HF.

In this work, we used CR-SPRC, a novel dosage form of SPRC by solid dispersion technique, to investigate the possible cardioprotective effects of it on HF rats. Compared with normal SPRC, CR-SPRC showed better effects because of the natural property of sustained release. Considering the characters of hydrogen sulfide and chronic heart failure, we firmly believe CR-SPRC can be a safe and effective candidate for hydrogen sulfide-mediated long-term therapy in the future.

## Supporting Information

Figure S1 **CR-SPRC preserved oxidative stress and prevented cell damage.**
(DOCX)

Figure S2 **CR-SPRC inhibited activity of caspases.**
(DOCX)

Figure S3 **SPRC protected against apoptosis in H9c2 cell lines.**
(DOCX)

Figure S4 **Carrier Eudragit prolonged the release of its contents SPRC** ***in vitro*** **.**
(DOCX)

Figure S5 **Carrier Eudragit prolonged the release of its contents SPRC** ***in vivo*** **.**
(DOCX)

Methods S1 **Supplemental methods.**
(DOCX)
