# Supplementary material for: Cardioprotective Effects of a Novel Hydrogen Sulfide Agent–Controlled Release Formulation of S-Propargyl-Cysteine on Heart Failure Rats and Molecular Mechanisms
Source: PLoS One. 2013 Jul 9;8(7):e69205. doi: 10.1371/journal.pone.0069205 (PMC3706411; doi:10.1371/journal.pone.0069205)
Supplement: Figure S2 — CR-SPRC inhibited activity of caspases. (DOCX) [file pone.0069205.s002.docx]

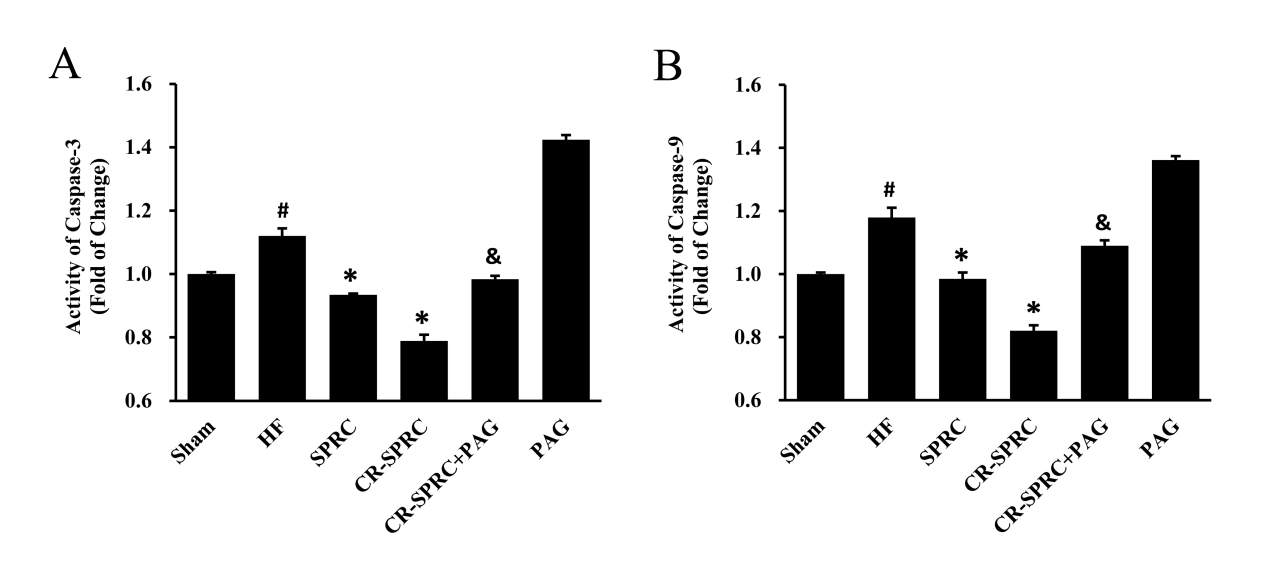


**Figure S2. CR-SPRC inhibited activity of caspases.**

The levels of caspase 3 and caspase 9 in plasma were determined by colorimetry, and statistically analyzed. Data were presented as means ± standard deviations (n=10). ^#^*P*<0.01 versus sham, ^*^*P*<0.01 versus HF, ^&^*P*<0.01 versus CR-SPRC. All experiments repeated at least 3 times.
