# Supplementary material for: Cardioprotective Effects of a Novel Hydrogen Sulfide Agent–Controlled Release Formulation of S-Propargyl-Cysteine on Heart Failure Rats and Molecular Mechanisms
Source: PLoS One. 2013 Jul 9;8(7):e69205. doi: 10.1371/journal.pone.0069205 (PMC3706411; doi:10.1371/journal.pone.0069205)
Supplement: Figure S3 — SPRC protected against apoptosis in H9c2 cell lines. (DOCX) [file pone.0069205.s003.docx]

**Figure S3. SPRC protected against apoptosis in H9c2 cell lines.**

The H9c2 cells were cultured in serum- and glucose-deficient DMEM, and then placed in an anaerobic GENBox for 8 hours to induce ischemia. SPRC (100 μmol/L) or PAG (1 mmol/L) was added at the same time as the model was induced. The cells in control group were still cultured with normal medium. The expression of Bax, Bad, Bcl-2, Bcl-xl, cleaved Caspase-3 and cleaved Caspase-9 were detected by Western blot. β-Tubulin was used as a loading control. All experiments repeated at least 3 times.
