# Supplementary material for: Cardioprotective Effects of a Novel Hydrogen Sulfide Agent–Controlled Release Formulation of S-Propargyl-Cysteine on Heart Failure Rats and Molecular Mechanisms
Source: PLoS One. 2013 Jul 9;8(7):e69205. doi: 10.1371/journal.pone.0069205 (PMC3706411; doi:10.1371/journal.pone.0069205)
Supplement: Figure S4 — Carrier Eudragit prolonged the release of its contents SPRC in vitro . (DOCX) [file pone.0069205.s004.docx]

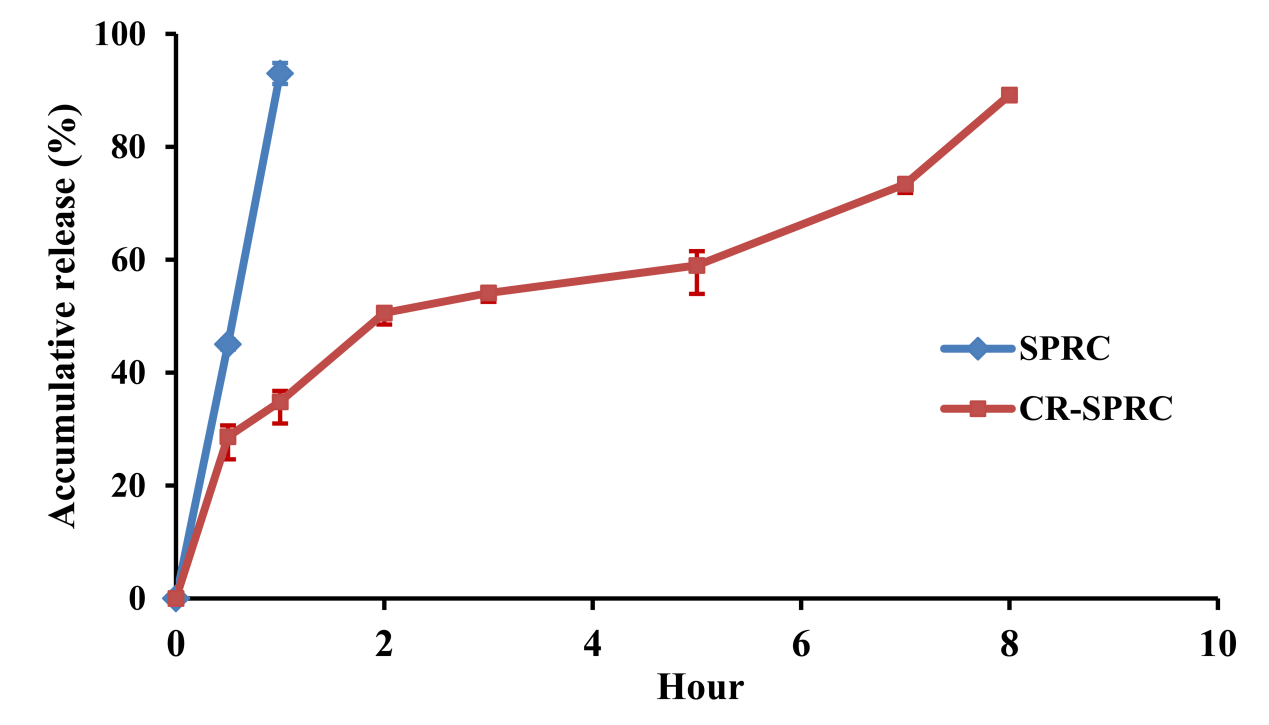


**Figure S4. Carrier Eudragit prolonged the release of its contents SPRC *in vitro*.**

The *in vitro* release rate of SPRC from the solid dispersions was measured using the paddle method. Data were presented as means ± standard deviations. The experiment repeated three times.
