# Supplementary material for: Cardioprotective Effects of a Novel Hydrogen Sulfide Agent–Controlled Release Formulation of S-Propargyl-Cysteine on Heart Failure Rats and Molecular Mechanisms
Source: PLoS One. 2013 Jul 9;8(7):e69205. doi: 10.1371/journal.pone.0069205 (PMC3706411; doi:10.1371/journal.pone.0069205)
Supplement: Figure S5 — Carrier Eudragit prolonged the release of its contents SPRC in vivo . (DOCX) [file pone.0069205.s005.docx]

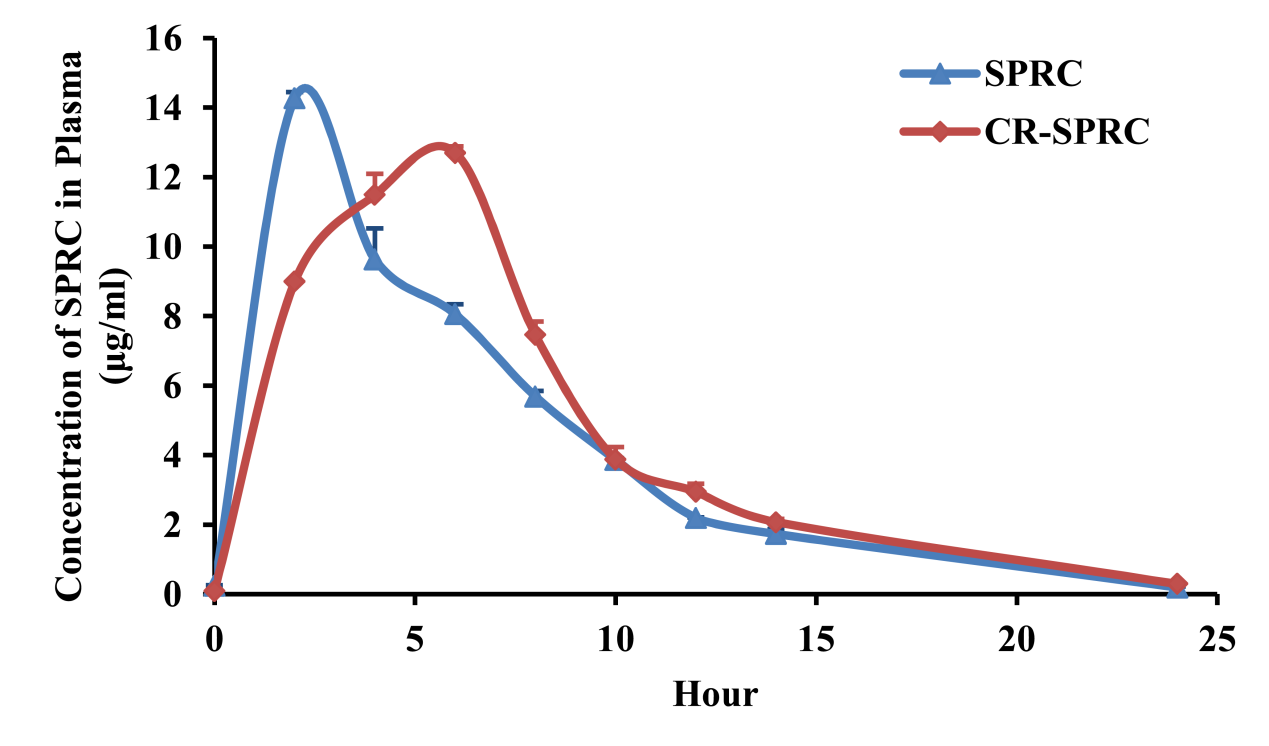


**Figure S5. Carrier Eudragit prolonged the release of its contents SPRC *in vivo*.**

The rats were divided randomly into 2 groups, and treated with SPRC or CR-SPRC. Then the blood was collected at different indicated time point, and centrifuged to get plasma. The levels of SPRC in plasma were determined using HPLC-MS/MS. Data were presented as means ± standard deviations (n=6).
