# Supplementary material for: Cardioprotective Effects of a Novel Hydrogen Sulfide Agent–Controlled Release Formulation of S-Propargyl-Cysteine on Heart Failure Rats and Molecular Mechanisms
Source: PLoS One. 2013 Jul 9;8(7):e69205. doi: 10.1371/journal.pone.0069205 (PMC3706411; doi:10.1371/journal.pone.0069205)
Supplement: Methods S1 — Supplemental methods. (DOCX) [file pone.0069205.s006.docx]

**Supplemental Methods**

*Cell culture and simulated ischemia model*

H9c2 cardiac myoblastic cell line was obtained from ATCC (Manassas, VA), and cultured in DMEM (Gibco, Grand Island, NY) with 10% FBS (Hyclone, Logan, UT) and 1% Penicillin Streptomycin (Gibco). As previously reported [[1](#_ENREF_1)], ischemia model was induced by using GENbox (BioMérieux, Marcy l'Etoile, France), which catalytically reduced O_2_ concentration to 1% within 30 min at 37 °C, and besides, replacing the medium to serum- and glucose-deficient DMEM (Gibco) to treat the cells for 8 hours. The drugs were added at the same time as the model was induced.

*Cell damage and oxidative stress assay*

The measurement of creatine kinase (CK), glutathione(GSH), catalase (CAT) and superoxide dismutase (SOD) in plasma was conducted by using commercially available kits according to the manufacturer’s instructions (Jiancheng Bioengineering Institute, Nanjing, China).

*Determination of Caspases*

Activity of caspase 3 and caspase 9 in plasma was determined by colorimetric assay using a microplate reader at 400nm. The assay kits were purchased from Biovision (Milpitas, CA).

*Western blot*

The proteins of H9c2 cells were extracted by ice-cold RIPA buffer (Pierce, Pittsburgh, PA). After boiling with loading buffer (Fermentas, Glen Burnie, MD), denatured proteins were separated in SDS-PAGE gel, and transferred onto PVDF membrane. The membrane was blocked with nonfat milk, followed by incubation with primary antibody of Bax, Bad, Bcl-2, Bcl-xl, cleaved Caspase-3 and cleaved Caspase-9 (Cell Signaling Technology, Boston, MA) at 4 °C overnight. HRP-conjugated secondary antibody (Kangchen Bio-tech, Beijing, China) was used to incubate the membrane for another 1 hour the next day. Immobilon™ Western Chemiluminescent HRP Substrate (Millipore, Billerica, MA) was poured on the membrane to develop the band captured by FluorChem Image System (Alpha Innotech, Santa Clara, California).

*In vitro release profile*

The release rate of SPRC from the solid dispersions was measured in a dissolution apparatus type DISTEK 2100B, using the paddle method (ChP II method). In each dissolution vessel, quantities of solid dispersions equivalent to 0.5 g of SPRC or CR-SPRC were placed. The test was performed at 37 ± 0.5 °C with a rotation speed of 50 rpm. The dissolution medium was 500 ml of 0.1 M hydrochloric acid. At predetermined time intervals, 5 ml of samples was withdrawn from the dissolution medium, filtered through 0.22μm membranes and assayed spectrophotometrically for the drug at 220 nm. An equal volume of fresh dissolution medium was transferred to the flask after sample withdrawal.

*Determination of SPRC by HPLC-MS/MS*

The rats were divided randomly into 2 groups (n=6), and treated with SPRC or CR-SPRC (30mg∙kg^-1^day^-1^). Then the blood was collected at different indicated time point in 24 h period, and centrifuged to get plasma. The levels of SPRC in plasma were determined using HPLC-MS/MS according to the previous protocol [[2](#_ENREF_2)].

**References**

1. Ekhterae D, Lin Z, Lundberg MS, Crow MT, Brosius FC, et al. (1999) ARC inhibits cytochrome c release from mitochondria and protects against hypoxia-induced apoptosis in heart-derived H9c2 cells. Circ Res 85: e70-e77.

2. Zheng Y, Liu H, Ma G, Yang P, Zhang L, et al. (2011) Determination of S-propargyl-cysteine in rat plasma by mixed-mode reversed-phase and cation-exchange HPLC–MS/MS method and its application to pharmacokinetic studies. J Pharm Biomed Anal 54: 1187-1191.
